# Cocaine regulation of *Nr4a1* chromatin bivalency and mRNA in male and female mice

**DOI:** 10.1038/s41598-022-19908-9

**Published:** 2022-09-21

**Authors:** Delaney K. Fischer, Keegan S. Krick, Chloe Han, Morgan T. Woolf, Elizabeth A. Heller

**Affiliations:** 1 Neuroscience Graduate Group, University of Pennsylvania, Philadelphia, PA USA; 2 Cell and Molecular Biology Graduate Group, University of Pennsylvania, Philadelphia, PA USA; 3 College of Arts & Sciences, University of Pennsylvania, Philadelphia, PA USA; 4 Institute for Translational Medicine and Therapeutics, University of Pennsylvania, Philadelphia, Pennsylvania USA; 5 Penn Epigenetics Institute, Perelman School of Medicine, University of Pennsylvania, Philadelphia, PA USA; 6 Department of Systems Pharmacology and Translational Therapeutics, University of Pennsylvania, Philadelphia, PA USA

**Keywords:** Molecular neuroscience, Epigenetics in the nervous system, Reward

## Abstract

Cocaine epigenetically regulates gene expression via changes in histone post-translational modifications (HPTMs). We previously found that the immediate early gene *Nr4a1* is epigenetically activated by cocaine in mouse brain reward regions. However, few studies have examined multiple HPTMs at a single gene. Bivalent gene promoters are simultaneously enriched in both activating (H3K4me3 (K4)) and repressive (H3K27me3 (K27)) HPTMs. As such, bivalent genes are lowly expressed but poised for activity-dependent gene regulation. In this study, we identified K4&K27 bivalency at *Nr4a1* following investigator-administered cocaine in male and female mice. We applied sequential chromatin immunoprecipitation and qPCR to define *Nr4a1* bivalency and expression in striatum (STR), prefrontal cortex (PFC), and hippocampus (HPC). We used Pearson’s correlation to quantify relationships within each brain region across treatment conditions for each sex. In female STR, cocaine increased *Nr4a1* mRNA while maintaining *Nr4a1* K4&K27 bivalency. In male STR, cocaine enriched repressive H3K27me3 and K4&K27 bivalency at *Nr4a1* and maintained *Nr4a1* mRNA. Furthermore, cocaine epigenetically regulated a putative NR4A1 target, *Cartpt*, in male PFC. This study defined the epigenetic regulation of *Nr4a1* in reward brain regions in male and female mice following cocaine, and, thus, shed light on the biological relevance of sex to cocaine use disorder.

## Introduction

Despite decades of research, cocaine use disorder remains a global health problem for which there is currently no FDA approved treatment. Despite clear epidemiological evidence that cocaine abuse afflicts men and women differently^1^, the underlying neurobiology of how cocaine impacts each sex is not fully understood. Human sex differences in behavior include higher initiation of cocaine use by men than women, a greater percent of men addicted to cocaine than women^2^, and a faster shift from casual drug use to addiction in women than men^3^. Additionally, women enter treatment at an earlier time point than men, have a higher relapse rate^3^, both of which may reflect women being more vulnerable to cue-induced relapse than men^4^. Importantly, rodent models of cocaine abuse recapitulate sexually dimorphic behavior, such that female rodents are more responsive to drug-conditioned stimuli^5,6^ and acquire cocaine-self administration at a faster rate than male rodents^7,8^. Recent research has indicated there are sexually dimorphic molecular effects of cocaine that might mediate behavioral phenotypes. For example, it was recently shown that cocaine broadly regulates distinct sets of proteins in males relative to females following cocaine self-administration in rodents^9^. Given the prominent sexually dimorphic cocaine-associated behavioral phenotypes and the distinct neuroadaptations for males and females^10^, sex must be considered a biological variable within drug research.

On a molecular level, cocaine regulates gene expression in part through changes in histone post-translational modifications (HPTMs). Such changes can be transient or persist across long periods of abstinence^11–18^. HPTMs tune gene expression in large part by altering chromatin accessibility and recruiting transcriptional machinery^13^. Acute and chronic cocaine impact HPTMs in multiple brain reward regions^17,19,20^, such as the striatum (STR)^18,21^, hippocampus (HPC)^22–24^, and prefrontal cortex (PFC)^25,26^. However, the complexities of chromatin remodeling following cocaine has yet to be explored in both sexes.

The histone code has historically proposed that engagement of individual HPTMs at a chosen locus can induce changes in protein recruitment and impact gene expression. However, the code also posits that HPTMs can be interdependent and/or work in tandem to impact gene expression^27,28^. Thus, this model suggests that chromatin accessibility and, correspondingly, gene expression are largely determined not only by the concentration of single HPTMs at a given gene promoter, but also by the combination of co-localized HPTMs^27^.

While many studies demonstrate genome-wide regulation of individual HPTMs following cocaine exposure^13,29–31^, the literature lacks investigation of how cocaine impacts co-localization of HPTMs. Bivalent gene promoters are comprised of co-localized activating (H3K4me3 (K4)) and repressive (H3K27me3 (K27)) HPTMs^32^ at a single promoter. H3K4me3 and H3K27me3 may be localized to discrete nucleosomes within the same promoter, or co-exist on the same nucleosome or H3 tail^33^. The presence of both modifications results in a poised state of gene expression, which can be ‘resolved’ to an active or repressive state by the removal of either H3K27me3 or H3K4me3 in response to transcriptional cues^32^. Bivalency may also protect against spurious and irreversible silencing of promoters by DNA hypermethylation, maintaining the epigenetic flexibility of stimuli-responsive genes^34^. While limited studies have investigated bivalency in the brain^35–37^, none to date have specifically examined bivalency with regard to sex or cocaine exposure.

We, and others, recently discovered that cocaine exposure activates expression of nuclear receptor subfamily 4 group A member 1 (*Nr4a1*) in the nucleus accumbens (NAc), a part of the STR^12,38,39^. Studies show that activation of *Nr4a1* in the NAc attenuates cocaine self-administration^38^, cocaine conditioned place preference^38^, cocaine sensitization^40^, and amphetamine-induced locomotion^41^. *Nr4a1* functions as a transcription factor at target genes involved in drug reward, such as *Cocaine- and amphetamine-regulated transcript* (*Cartpt), Tyrosine Hydroxylase (Th), solute carrier family 6 member 3* (DAT production)*,* and Brain-derived neurotrophic factor (*Bdnf)*^38,42,43^*,* as well as plasticity in reward-related brain regions^40,44^. However, the precise function of HPTMs in cocaine-induced *Nr4a1* expression has yet to be elucidated. Additionally, there is conspicuous lack of data on the regulation of *Nr4a1* where sex is a defined variable. Accordingly, we investigated K4&K27 bivalency in the cocaine regulation of *Nr4a1* expression in both male and female mice.

In this study, we utilized single sample sequencing (S3EQ)^45^ and sequential chromatin immunoprecipitation (ChIP)^35,46^ to independently investigate the transcriptomic and epigenomic profiles of *Nr4a1* from three different brain regions in males and females. We also utilized Pearson’s correlation matrices to capture the individual variability in biological changes induced by cocaine exposure in males and females.

## Results

### K4&K27 bivalency at *Nr4a1* promoter in brain reward regions was present in both male and female mice

**Figure 1:**
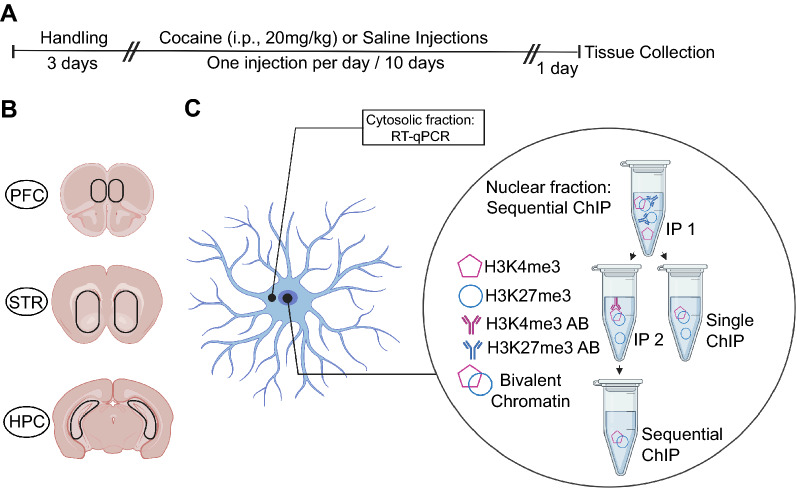
Methods used to investigate regional transcriptional and epigenomic profiles following cocaine exposure. (**A**) Timeline for investigator-administered cocaine (20 mg/kg; i.p) paradigm. (**B**) Coronal brain slice illustrations showing the brain regions taken for single sample sequencing (Top to bottom: prefrontal cortex (PFC), striatum (STR), hippocampus (HPC)). (**C**) Schematic showing the sequential ChIP protocol that includes two immunoprecipitations.

For epigenetic profiling of three reward-associated brain regions following cocaine treatment, we applied sequential ChIP to examine K4&K27 bivalency at *Nr4a1* (Fig. 1). ChIP-qPCR and ChIP-Seq protocols that quantify HPTM deposition (the addition of the modification) at monovalent promoters are unable to quantify combinatorial HPTM deposition at single promoter. This weakness stems from the fact that individual HPTM profiling experiments cannot distinguish between bivalent promoter deposition of two HPTMs in a single population of cells or monovalent promoter deposition of two HPTMs from a mixed population of cells^33^. The sequential ChIP protocol is required to define true bivalency of H3K4me3 and H3K27me3 deposition at the same locus in a single population of cells (and nucleosomes)^33^. To measure bivalency, we split each single sample and performed H3K4me3 ChIP followed by H3K27me3 ChIP. From the same sample, we also performed H3K27me3 ChIP followed by H3K4me3 ChIP. In this way, sequential ChIP was assayed with either H3K4me3 or H3K27me3 as the first IP, and the other HPTM as the second IP. This approach allowed us to interpret differences in cocaine-regulated K4&K27 bivalency due to differences in rates of deposition or removal between the two HPTMs^47^. In each case, enrichment based on antibody order was quantified by qCHIP. As we found no differences in results of qCHIP by order of sequential ChIP, we concluded that both approaches demonstrated *Nr4a1* K4&K27 promoter bivalency.

### Cocaine enriched H3K27me3 and bivalency at the *Nr4a1 *promoter and did not alter *Nr4a1* mRNA levels in male striatum

**Figure 2:**
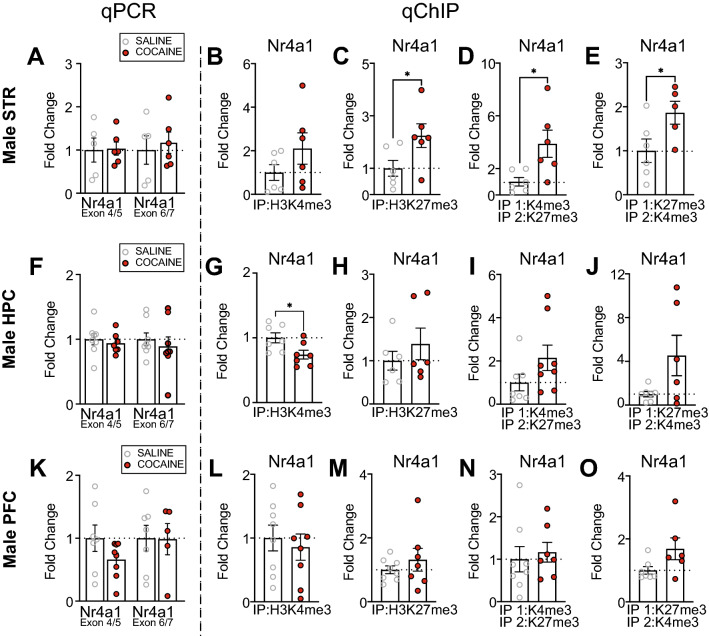
Cocaine alters the epigenetic profile of *Nr4a1* in the striatum and hippocampus of male mice. (**A**) No change in mRNA levels of *Nr4a1* (as examined by primer pairs that cover the junction between exon 4/5 and exon 6/7) were observed in the male mouse STR following 10 days of investigator-administered cocaine (n = 5–6/group). There was no change in (**B**) H3K4me3 (n = 6/group), but an increase in (**C**) H3K27me3 (n = 6/group), (**D**) H3K4me3/H3K27me3 bivalency (n = 6/group), and (**E**) H3K27me3/H3K4me3 bivalency (n = 5–6/group) at *Nr4a1* in the male mouse STR following 10 days of investigator-administered cocaine. (**F**) There was no change in mRNA levels of *Nr4a1* (as measured by independent primer pairs that cover the junction between exons 4/5 and exons 6/7) in the male mouse HPC following 10 days of investigator-administered cocaine (n = 7–8/group). There was a decrease in (**G**) H3K4me3 at *Nr4a1* (n = 7/group), but no change in (**H**) H3K27me3 (n = 6/group), (**I**) H3K4me3/H3K27me3 bivalency (n = 7–8/group), or (**J**) H3K27me3/H3K4me3 bivalency (n = 6–7/group) at *Nr4a1* in the male mouse HPC following 10 days of investigator-administered cocaine. (**K**) There was no change in mRNA levels of *Nr4a1* (as measured by independent primer pairs that cover the junction between exons 4/5 and exons 6/7) in the male mouse PFC following 10 days of investigator-administered cocaine (n = 7–8/group). There is no change in (**L**) H3K4me3 (n = 8/group), (**M**) H3K27me3 (n = 7–8/group), (**N**) H3K4me3/H3K27me3 bivalency (n = 7–8/group), or (**O**) H3K27me3/H3K4me3 bivalency at *Nr4a1* in the male mouse PFC following 10 days of investigator-administered cocaine (n = 6–7/group). *p < 0.05. Data are shown as mean ± SEM. STR, striatum; HPC, hippocampus; PFC, prefrontal cortex.

Following cocaine treatment, there was no change in *Nr4a1* mRNA (Fig. 2A) or H3K4me3 deposition at *Nr4a1* in the male STR (Fig. 2B). In the same samples, there was increased H3K27me3 at *Nr4a1* (Fig. 2C) (t_10_ = 2.3, *p* = 0.0443) and an increase in K4&K27 bivalency at the *Nr4a1* promoter (Fig. 2D,E) (**D**: t_5.973_ = 2.663, *p* = 0.0376; **E**: t_9_ = 2.298, *p* = 0.0476). In the male HPC following cocaine treatment, there was no change in *Nr4a1* mRNA (Fig. 2F), but we observed depletion of H3K4me3 at *Nr4a1* (Fig. 2G) (t_12_ = 2.528, *p* = 0.0265). In the same samples, there was no change in H3K27me3 deposition or K4&K27 bivalency (Fig. 2H,I,J). In the male PFC following cocaine treatment, there was no change in *Nr4a1* mRNA (Fig. 2K), H3K4me3, H3K27me3, or K4&K27 bivalency (Fig. 2L–O).

### Cocaine increased Cartpt mRNA in PFC of male mice

**Figure 3:**
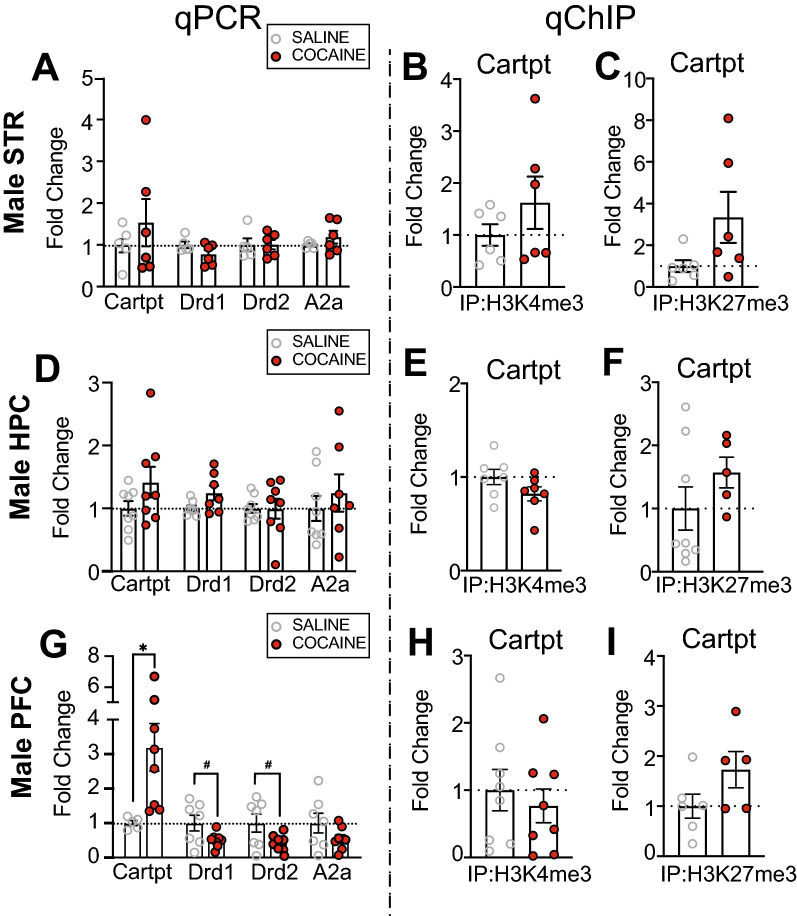
*Nr4a1* downstream target, *Cartpt*, is regionally activated after 10 days of investigator-administered cocaine in male mice. (**A**) There was no change in mRNA levels of *Cartpt, Drd1, Drd2,* or *A2a* in the male mouse STR following 10 days of investigator-administered cocaine (n = 5–6/group). There was no change in (**B**) H3K4me3 (n = 6/group) or (**C**) H3K27me3 (n = 6/group) at *Cartpt* in the male mouse STR after 10 days of investigator-administered cocaine. (**D**) There was no change in mRNA levels of *Cartpt, Drd1, Drd2,* or *A2a* in the male mouse HPC following 10 days of investigator-administered cocaine (n = 7–8/group). There was no change in (**E**) H3K4me3 (n = 7/group) or (**F**) H3K27me3 (n = 6–8/group) at *Cartpt* in the male mouse HPC after 10 days of investigator-administered cocaine. (**G**) There was an increase in mRNA levels of *Cartpt,* a trending decrease in mRNA levels of *Drd1* and *Drd2,* and no change in mRNA levels of *A2a* in the male mouse PFC following 10 days of investigator-administered cocaine (n = 5–6/group). There was no change in (**H**) H3K4me3 (n = 8/group) or (**I**) H3K27me3 at *Cartpt* in the male mouse PFC after 10 days of investigator-administered cocaine (n = 5–6/group). *p < 0.05. ^#^*p* < 0.10. Data are shown as mean ± SEM. STR, striatum; HPC, hippocampus; PFC, prefrontal cortex.

Given the transient nature of immediate early gene expression, we also measured mRNA and HPTM deposition of a downstream transcriptional target of *Nr4a1*^38^. We found no changes in *Cartpt* mRNA, or in mRNA of dopamine receptors, *Drd1* and *Drd2*, or adenosine A2a receptor (*A2a*), in the male STR following cocaine treatment (Fig. 3A). Likewise, we observed no change in H3K4me3 or H3K27me3 deposition at *Cartpt* (Fig. 3B,C). Similarly, in the male HPC, cocaine treatment did not alter *Cartpt, Drd1, Drd2,* or *A2a* mRNA (Fig. 3D), or H3K4me3 or H3K27me3 deposition at *Cartpt* (Fig. 3E,F). Alternatively, in the male PFC, cocaine treatment increased *Cartpt* mRNA (t_7.165_ = 3.182, *p* = 0.0150), and we observed a trending decrease of *Drd1* mRNA (trending, t_7.247_ = 2.059, *p* = 0.0771), *Drd2* (trending, t_7.318_ = 2.110, *p* = 0.0711), but cocaine did not alter *A2a* mRNA levels (Fig. 3G). This was accompanied by no change in H3K4me3 or H3K27me3 deposition at *Cartpt* (Fig. 3H,I). These data show that cocaine induces *Cartpt* in the male PFC.

### Cocaine increased *Nr4a1* mRNA in multiple reward-associated brain regions in female mice

**Figure 4:**
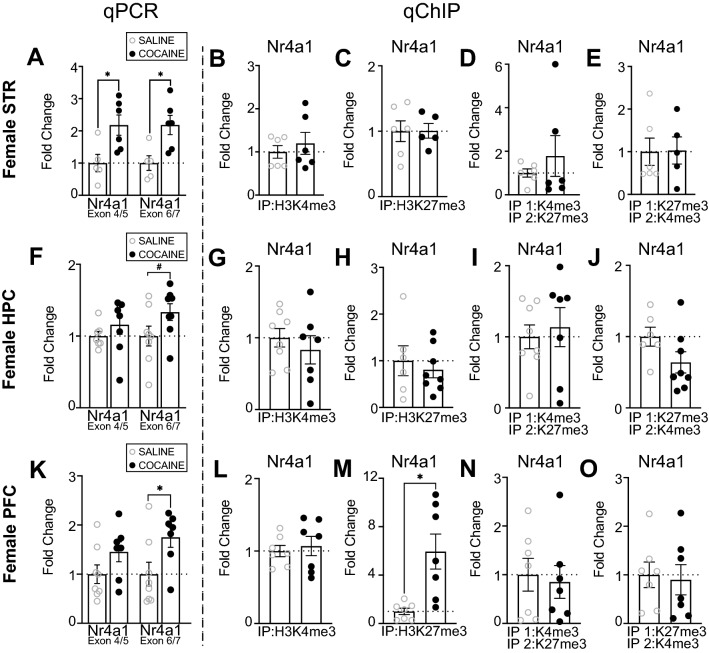
Cocaine alters the transcriptomic profile of *Nr4a1* in the STR of females and the transcriptomic and epigenetic profile of *Nr4a1* in the PFC of females. (**A**) There was an increase in mRNA levels of *Nr4a1* (as examined by primer pairs that cover the junction between exons 4/5 and exons 6/7) in the female mouse STR following 10 days of investigator-administered cocaine (n = 4–6/group). There was no change in (**B**) H3K4me3 (n = 6/group), (**C**) H3K427me3 (n = 5–6/group), (**D**) H3K4me3/H3K27me3 bivalency (n = 6/group)**,** or (**E**) H3K27me3/H3K4me3 bivalency (n = 5–6/group) at *Nr4a1* in the female mouse STR after 10 days of investigator-administered cocaine. (**F**) There was no change in mRNA levels of *Nr4a1* as measured by independent primer pairs that cover the junction between exons 4/5, and a trending increase in mRNA levels of *Nr4a1* as measured by independent primer pairs that cover the junction between exons 6/7 in the female mouse HPC following 10 days of investigator-administered cocaine (n = 7–8/group). There was no change in (**G**) H3K4me3 (n = 7–8/group), (**H**) H3K27me3 (n = 6–8/group), (**I**) H3K4me3/H3K27me3 (n = 7–8/group), or (**J**) H3K27me3/H3K4me3 bivalency (n = 6–8/group) at *Nr4a1* in the female mouse HPC following 10 days of investigator-administered cocaine. (**K**) There was an increase in mRNA levels of *Nr4a1* (as measured by independent primer pairs that cover the junction between exons 6/7, but not exons 4/5) in the female mouse PFC following 10 days of investigator-administered cocaine (n = 7–8/group). There was no change in (**L**) H3K4me3 (n = 7/group), but there was an increase in (**M**) H3K27me3 (n = 7/group) at *Nr4a1* following 10 days of investigator-administered cocaine in the female mouse PFC (n = 7/group). (**N**) There was no change in H3K4me3/H3K27me3 bivalency (n = 7/group) or (**O**) H3K27me3/H3K4me3 bivalency at *Nr4a1* in the female mouse prefrontal cortex following 10 days of investigator-administered cocaine (n = 7/group). *p < 0.05. ^#^*p* < 0.10. Data are shown as mean ± SEM. STR, striatum; HPC, hippocampus; PFC, prefrontal cortex.

Following cocaine treatment, *Nr4a1* mRNA was increased in the female STR (Fig. 4A). This increase was independently identified using two different primer sets, each of which spans the junction between two distinct exons within *Nr4a1* (*Nr4a1* Exon 4/5: t_9_ = 2.771, *p* = 0.0217; *Nr4a1* Exon 6/7: t_9_ = 3.030, *p* = 0.0142). In the same samples, there was no change in H3K4me3, H3K27me3, or K4&K27 bivalency at *Nr4a1* (Fig. 4B–E). In the female HPC, there was no change in *Nr4a1* mRNA following cocaine treatment when the *Nr4a1* Exon 4/5 primer set was used, but we observed a trending increase in *Nr4a1* mRNA when mRNA levels were evaluated with Exon 6/7 primers (trending, t_14_ = 1.1841, *p* = 0.0869) (Fig. 4F). In the same samples, there was no change in *Nr4a1* H3K4me3, H3K27me3 or K4&K27 bivalency (Fig. 4G–J). In the female PFC following cocaine treatment, *Nr4a1* mRNA was increased when measured at *Nr4a1* E6/7 but not *Nr4a1* E4/5 (*Nr4a1* Exon 6/7: t_13_ = 2.327, *p* = 0.0368) (Fig. 4K). In the same samples, there was no change in H3K4me3 (Fig. 4L), there was H3K27me3 recruitment (t_6.392_ = 3.363, *p* = 0.0138) (Fig. 4M), and no change in K4&K27 bivalency (Fig. 4N,O). In sum, these data showed that cocaine treatment in female mice increased *Nr4a1* mRNA expression in the STR along with maintenance of H3K4me3, H3K27me3, and K4&K27 bivalency at the *Nr4a1* promoter. These data also showed that cocaine treatment in female mice increased *Nr4a1* mRNA expression in the PFC, despite recruitment of H3K27me3.

### Cocaine did not alter mRNA of drug-related markers, Cartpt mRNA, or Cartpt HPTMs in female mouse brain regions

**Figure 5:**
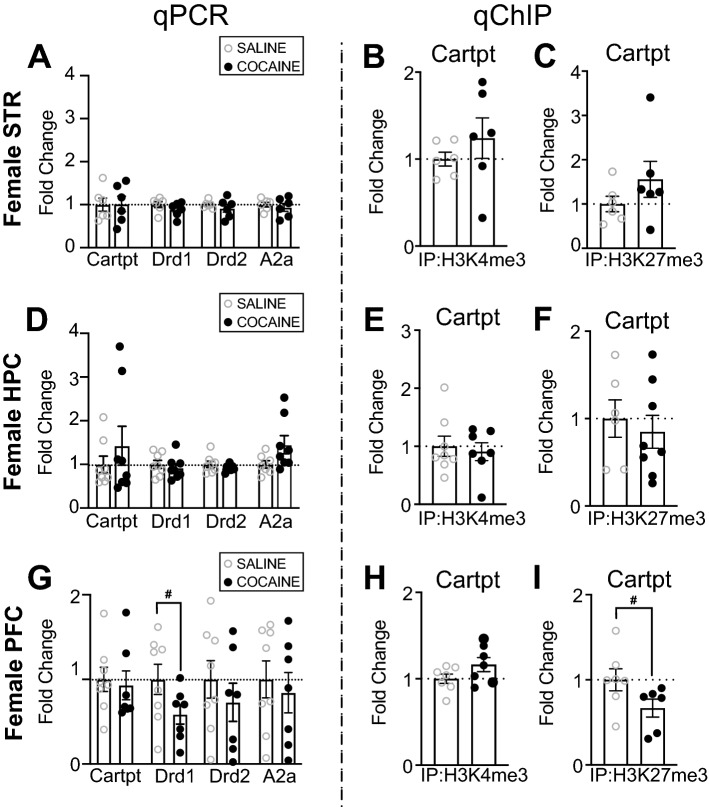
*Nr4a1* downstream target, *Cartpt*, and other drug-related markers are impacted after 10 days of investigator-administered cocaine in the PFC of female mice. (**A**) There was no change in mRNA levels of *Cartpt, Drd1, Drd2,* or *A2a* in the female mouse STR following 10 days of investigator-administered cocaine (n = 6/group). There was no change in (**B**) H3K4me3 (n = 6/group) or (**C**) H3K27me3 (n = 6/group) at *Cartpt* in the female mouse STR after 10 days of investigator-administered cocaine. (**D**) There was no change in mRNA levels of *Cartpt, Drd1, Drd2,* or *A2a* in the female mouse HPC following 10 days of investigator-administered cocaine (n = 8/group). There was no change in (**E**) H3K4me3 (n = 7–8/group) or (**F**) H3K27me3 (n = 6–8/group) at *Cartpt* in the female mouse HPC after 10 days of investigator-administered cocaine. (**G**) There was a trending decrease in mRNA levels of *Drd1* and no change in mRNA levels of *Cartpt, Drd2,* or *A2a* in the female mouse PFC following 10 days of investigator-administered cocaine (n = 7–8/group). There was no change in (**H**) H3K4me3 (n = 7–8/group) but there was a trending decrease in (**I**) H3K27me3 at *Cartpt* in the female mouse PFC after 10 days of investigator-administered cocaine (n = 7–8/group). *p < 0.05. ^#^*p* < 0.10. Data are shown as mean ± SEM. STR, striatum; HPC, hippocampus; PFC, prefrontal cortex.

As we saw independent increases in mRNA levels of *Nr4a1* following cocaine in female mice in the PFC and the STR, we expected to find transcriptomic or epigenetic changes at *Cartpt*, a downstream target of *Nr4a1*^38^. Surprisingly, we found no transcriptomic or epigenetic changes (H3K4me3, H3K27me3) at *Cartpt* in female mice following cocaine in the STR (Fig. 5A–C), HPC (Fig. 5D–F), or PFC (Fig. 5G–I), although we observed trending depletion of H3K27me3 levels at *Cartpt* in the PFC (Fig. 5I) (t_11_ = 1.953, *p* = 0.0767**).** Notably, we also observed no change in mRNA levels of *Drd1*, *Drd2*, or *A2a* in the STR, HPC, or PFC (Fig. 5A,D,G) in female mice following cocaine, but observed a trending decrease in mRNA levels of *Drd1* in the PFC (Fig. 5G) (t_13_ = 1.887, *p* = 0.0818).

### Cocaine impacted correlational relationships within and between the transcriptomic and epigenetic profiles of Nr4a1 and Cartpt in male and female mice

**Figure 6:**
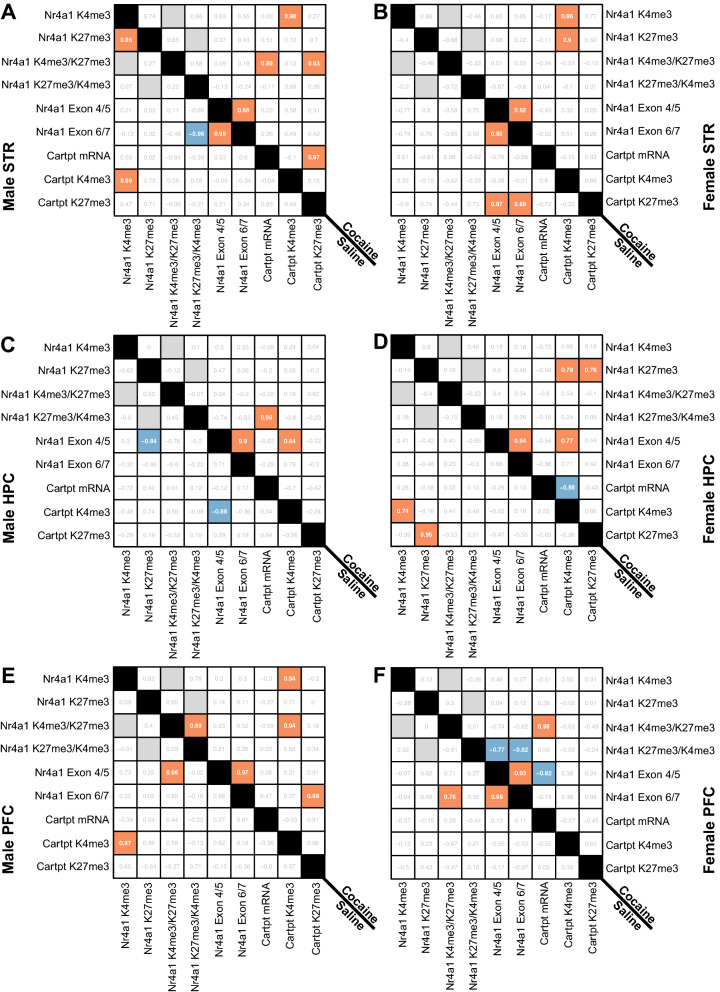
Pearson’s Correlation Matrices reveal distinct transcriptomic and epigenetic relationships following cocaine exposure in both sexes. (**A**) Pearson’s Correlation matrix evaluating transcriptomic and epigenetic relationships of *Nr4a1* and *Cartpt* in the striatum of saline- and cocaine-injected males and (**B**) females. (**C**) Pearson’s Correlation matrix evaluating transcriptomic and epigenetic relationships of *Nr4a1* and *Cartpt* in the hippocampus of saline- and cocaine-injected males and (**D**) females. (**E**) Pearson’s Correlation matrix evaluating transcriptomic and epigenetic relationships of *Nr4a1* and *Cartpt* in the prefrontal cortex of saline- and cocaine-injected males and (**F**) females. For all Pearson’s Correlation matrices**,** red indicates a significant positive correlation (*p* ≤ 0.05) and blue indicates a significant negative correlation (*p* ≤ 0.05). A gray box indicates a correlation was not analyzed as it violates the rule of independence for Pearson’s Correlation.

As we hypothesized that we would see a linear relationship between *Nr4a1* and *Cartpt* following cocaine, we utilized Pearson’s correlation matrices to explore the relationships within our sample population between the transcriptomic and epigenetic profiles of *Nr4a1* and *Cartpt.* Every significant correlation we observed can be found in Supplementary Tables 3–5 which also provides the corresponding figure(s) that show the data within each correlation in Fig. 6 (example: the data used to reveal the positive correlation between *Nr4a1* mRNA and *Cartpt* H3K27me3 in the STR of saline-treated females can been seen in Figs. 4A and 5C.) In the STR we observed positive correlations between (1) *Nr4a1* H3K4me3 and *Cartpt* H3K4me3 and (2) mRNA levels of *Nr4a1* measured by the two primers pairs spanning either Exon 4/5 or Exon 6/7 across treatments (saline, cocaine) and sex (male, female) (Fig. 6A,B). In the STR of saline-treated females, we observed a positive correlation between (3) *Nr4a1* mRNA and *Cartpt* H3K27me3 (Fig. 6B). In the STR of males following cocaine, we observed a positive correlation between (4) *Cartpt* mRNA and *Cartpt* H3K27me3, (5) *Cartpt* mRNA and *Nr4a1* K4/K27 bivalency, and (6) *Cartpt* H3K27me3 and *Nr4a1* K4/K27 bivalency (Fig. 6A). In the STR of females following cocaine, we observed a positive correlation between (7) *Nr4a1* H3K4me3 and H3K4me3 at *Cartpt* and (8) *Nr4a1* H3K27me3 and *Cartpt* H3K4me3 (Fig. 6B).

In the HPC of saline-treated male mice we observed a negative correlation between (1) *Nr4a1* mRNA levels (Exon 4/5) and *Nr4a1* H3K27me3 and (2) *Nr4a1* mRNA levels (Exon 4/5) and *Cartpt* H3K4me3 (Fig. 6C). In the HPC of saline-treated females we observed a positive correlation between (3) *Nr4a1* H3K4me3 and *Cartpt* H3K4me3 and (4) *Nr4a1* H3K27me3 and *Cartpt* H3K27me3 (Fig. 6D). In the HPC of males following cocaine, we observed a positive correlation between (5) *Nr4a1* mRNA levels (Exon 4/5 and Exon 6/7), (6) *Nr4a1* mRNA levels (Exon 4/5) and *Cartp*t H3K4me3, and (7) *Cartpt* mRNA levels and *Nr4a1* K27/K4 bivalency (Fig. 6C). In the HPC of females following cocaine, we observed a positive correlation between (8) *Nr4a1* mRNA levels (Exon 4/5 and Exon 6/7), (9) *Nr4a1* mRNA levels (Exon 4/5) and *Cartp*t H3K4me3, (10) *Nr4a1* H3K27me3 and *Cartpt* H3K4me3, and (11) *Nr4a1* H3K27me3 and *Cartpt* H3K27me3 (Fig. 6D). We observed a negative correlation in the HPC of females following cocaine between (12) *Cartpt* mRNA levels and *Cartpt* H3K4me3 (Fig. 6D).

In the PFC of saline-treated males, we found a positive correlation between (1) mRNA levels of *Nr4a1* (Exon 4/5) and H3K4me3/H3K27me3 at *Nr4a1* and (2) H3K4me3 at *Nr4a1* and H3K4me3 at *Cartpt* (Fig. 6E). In the PFC of saline-treated females, we found a positive correlation between (3) *Nr4a1* mRNA levels (Exon 4/5 and Exon 6/7) as well as (4) *Nr4a1* mRNA levels (Exon 6/7) and *Nr4a1* K4/K27 bivalency (Fig. 6F). In the PFC of cocaine-injected males, we found multiple positive correlations including: (5) H3K4me3/H3K27me3 bivalency at *Nr4a1* and H3K4me3 at *Cartpt*, (6) H3K4me3 at *Nr4a1* and H3K4me3 at *Cartpt*, (7) *Nr4a1* H3K4me3/H3K27me3 bivalency, (8) *Nr4a1* mRNA levels (Exon 4/5 and Exon 6/7), and (9) *Nr4a1* mRNA levels (Exon 6/7) and *Cartpt* H3K27me3 (Fig. 6E). In the PFC of females following cocaine, we observed both positive and negative correlations. We found that (10, 11) mRNA levels of *Nr4a1* (Exon 4/5 and Exon 6/7) negatively correlate with H3K27me3/H3K4me3 bivalency at *Nr4a1.* Additionally, (12) mRNA levels of *Nr4a1* (Exon 4/5) negatively correlate with mRNA levels of *Cartpt* (Fig. 6F). We observed a positive correlation between (13) mRNA levels of *Cartpt* and H3K4me3/H3K27me3 bivalency at *Nr4a1* and (14) mRNA levels of *Nr4a1* between its primer sets (Exon 4/5 and Exon 6/7) (Fig. 6F).

## Discussion

There is emerging literature on the transcriptomic and epigenetic profiles of *Nr4a1* and its regulation by cocaine. Despite this progress, no study to date has explored basal and cocaine-activated regulation of *Nr4a1* in both males and females in any organism. Additionally, few studies have assessed bivalency in the brain^35–37^, and none, to our knowledge, have investigated cocaine regulation of bivalency. Here, we demonstrate that, following cocaine, *Nr4a1* mRNA increases in female mouse STR and PFC, and does not change in male mouse STR, HPC, or PFC. We also find an increase in *Nr4a1* K4&K27 promoter bivalency in the male STR following cocaine treatment. Finally, we show that the mRNA level of a putative *Nr4a1* target, *Cartpt*, is activated in the male PFC.

Our results are the first to establish that there is a relationship between bivalency and cocaine in the brain, specifically we show induction of bivalency in the male STR. As this mechanism has not yet been linked to drugs of abuse, it provides a new mechanism of exploration for drug abuse research. Bivalent chromatin regulates DNA accessibility rather elegantly by transitioning euchromatin or heterochromatin to a poised, bivalent state that can quickly react to cellular cues. This flexibility is beneficial for cell differentiation^32^ and stress responses^35^, but may underlie detrimental instability of gene expression in the adult brain. Consequently, bivalency is implicated in transcriptional dysregulation observed in Huntington’s Disease^48^ as well as multiple types of cancer^49–52^. We hypothesize that bivalency may similarly underlie transcriptional dysregulation in drug addiction, in both males and females. Our data highlight that the interplay between bivalency and cocaine warrants further investigation.

The current study profiles both ventral and dorsal STR, as well as HPC and PFC, other brain regions associated with reward neuropathology. Both the ventral STR (NAc) and dorsal STR are involved in cocaine reinforcement, and the interconnectivity between these regions mediates cocaine-seeking behavior^53^. Additionally, pharmacological studies have shown that dopamine transporters in both the NAc and dorsal STR have the same affinity for cocaine binding^54^, and that the connectivity between dorsal STR and NAc is necessary for the regulation of dopamine to mediate cocaine’s reinforcing properties^55^. Our lab and others find that cocaine treatment increases *Nr4a1* mRNA in the NAc in mixed sex populations^38^, as well as in males and females when assessed separately^39^. However, in order to analyze K4&K27 bivalency at the *Nr4a1* promoter of a single mouse, it was necessary to isolate chromatin from combined STR and perform sequential ChIP. Both H3K4me3 and H3K27me3 are basally present at *Nr4a1*, with high enrichment for H3K4me3 in bulk tissue of the nucleus accumbens^31^ as well as in cell-type specific tissue of the combined striatum^56^, showing that combined STR and NAc have the same enrichment pattern. Additionally, both H3K4me3 and H3K27me3 are present at baseline at *Cartpt*, with high co-enrichment for both marks in bulk tissue of the nucleus accumbens^31^ and in cell-type specific tissue of the striatum^56^. Using sequential ChIP, we were able to identify the same patterns of enrichment at both modifications at baseline for *Nr4a1* and *Cartpt*.

The strengths of the sequential ChIP approach in deciphering the histone code are limited by the technical requirement of bulk STR starting material, which obscures cell-type specific *Nr4a1* regulation by cocaine and sex. The current literature contains complex and conflicting data on the expression pattern of *Nr4a1* in direct pathway, Drd1 + , and indirect pathway, Drd2 + and/or A2a + , medium spiny neurons (MSNs)^39,40,57,58^. Studies suggest that *Nr4a1* expression in Drd1 + MSNs, but not A2a + MSNs, functions in cocaine craving and relapse-like behavior. Drd1-specific signaling, such as the phosphoErk (pErk) cascade, increases in the NAc across incubation of cocaine craving^59^, and may regulate *Nr4a1* in the Drd1 + MSN subtype^40,57,59,60^.

Importantly, the relationships with bivalency (H3K4me3/H3K27me3 vs H3K27me3/H3K4me3) do not always align at the level of a correlation. This is likely inherent to the biological mechanism of bivalency. Induction of H3K4me3/H3K27me3 bivalency is indicative of a transition from an activating to poised state (bivalency results in lower activation), while induction of H3K27me3/H3K4me3 bivalency is indicative of a repressive to poised state (bivalency results in higher activation). Additionally, we found that *Nr4a1* mRNA levels did not always correspond with the canonical roles of H3K4me3, H3K27me3, and K4&K27 bivalency, in activating, repressing, and poising gene expression, respectively. For example, in the female PFC following cocaine treatment we observed concomitant increases in *Nr4a1* mRNA and H3K27me3 at *Nr4a1*. We thus applied correlational analyses to examine these relationships more comprehensively. In the male STR, we found a positive correlation between *Nr4a1* K4/K27 bivalency and Cartpt mRNA levels. Given that *Nr4a1* is a putative activator of *Cartpt* expression^38^, this finding went against our expectation that *Nr4a1* gene poising would be negatively correlated with *Cartpt* mRNA levels. Additionally, given the lack of transcriptomic or the epigenomic regulation of *Nr4a1* in the male PFC and the vast increase in *Cartpt* mRNA levels, we hypothesize that *Cartpt* may additionally be regulated by other distinct transcription factors and/or HPTMs following cocaine. This is supported by our correlational data that shows minimal overlap in the transcriptomic and epigenetic profiles of *Nr4a1* and *Cartpt* in either drug conditions in males or females or in any of the three regions examined. Another reason that some of the epigenetic and mRNA correlations may not have aligned could be due to the fact that our work focused solely on investigating *Nr4a1* bivalency at the promoter. Epigenetic investigation of other regulatory regions, such as enhancers, which influence transcription via signaling to promoters^61^, could shed further light onto epigenetic control of gene regulation. For example, H3K4me3 is predominantly located at promoters, although it has been also been identified as a modifier of enhancers^62^. Future studies will expand investigation of bivalency beyond promoters to other genomic regulatory regions, provided such regions have been identified and functionally validated.

Based on our findings and the literature, we also hypothesize that additional HPTMs, such as acetylation, regulate expression of *Nr4a1* and *Cartpt.* Intracranial HPC injection of the histone deacetylase (HDAC) inhibitor, Trichostatin A, increases *Nr4a1* mRNA and protein levels^63^, as does HDAC3 inhibition in the dorsal HPC^64^. In cell lines, *Nr4a1* expression is regulated by the recruitment of the histone acetyltransferase p300 or HDAC1^65^. It is interesting to consider that changes in STR histone acetylation, and methylation to a certain extent, are sensitive to the duration of cocaine treatment (acute versus chronic) and time since the last injection^21^, which may reflect the rapid turnover of acetylation, especially compared to methylation^47^. Although acetylation turnover is more rapid than methylation, we hypothesize that repeating the endpoints outlined in this study at a different time point, such as 30 min after the final injection versus 24 h, could prompt different levels of H3K4me3, H3K27me3, and K4&K27 bivalency. Specifically, we expect an initial decrease in H3K27me3 and K4&K27 bivalency (< 30 min following cocaine) followed by an increase in H3K27me3 and K4&K27 bivalency at later timepoints (> 24 h following cocaine).

Nonetheless, our findings encourage future studies to investigate combinatorial HPTMs beyond K4&K27 bivalency*.* For example, H3K27me3 and H3K27ac bivalency are well described in the literature and represent a tractable starting point for further exploration^66,67^. We hypothesize that cocaine-induced depletion of H3K27ac acts synergistically with increased K4&K27 bivalency to inhibit the activation of *Nr4a1* expression in males. We speculate that the persistence of H3K27me3 is sex-dependent, and thus H3K27me3 is likely bivalent with other HPTMs as well. This hypothesis is based in part on the sexually dimorphic expression of H3K27me3-demethylase, *Kdm6a*, in rodent brain^68–70^. As *Kdm6a* is X-linked^71^, it may mediate sex-specific changes in HPTM expression upon cocaine exposure to drive epigenetic changes at *Nr4a1* and other cocaine-responsive loci. It is possible that sexually dimorphic molecular changes underlie sexually dimorphic behaviors. For example, male rodents are less responsive to drug-conditioned stimuli^5,6^ and acquire cocaine-self administration at a slower rate than female rodents^7,8^. Future studies will explore additional combinatorial HPTMs with H3K27me3 to probe these hypotheses.

Additionally, new methods are being developed to allow for future studies to conduct sequential ChIP profiling of small amounts of input material which will allow for profiling of more specific brain regions and cell-types. For example, the recently developed multi-CUT&Tag^72^ allows for mapping HPTM co-localization in the same cell, and single-cell multi-CUT&Tag^72^ can probe for bivalency in specific cell types. While single-cell CUT&Tag has been carried out in mouse brain to profile individual HPTMs^73^, these multi-CUT&Tag methods that uncover bivalency have yet to be applied in brain tissue as far as we are aware. Finally, in the current study we test and support the hypothesis that K4&K27 bivalency is increased at *Nr4a1* in male mice following cocaine treatment, concomitant with increased H3K27me3 deposition. To test the sufficiency of loss of H3K27me3 in increased *Nr4a1* mRNA, we can apply in vivo epigenetic editing to exogenously enrich *Nr4a1* H3K27me3 in the presence of cocaine. This approach may also shed light onto the role of H3K27ac, given that dCas9-FOG1 interacts with the NuRD complex to cause HDAC1/2-mediated removal of H3K27ac^74^.

In closing, despite epidemiological evidence that cocaine abuse afflicts both men and women^1^, the underlying neurobiology of how cocaine impacts each sex is not fully understood. This is problematic as a growing body of knowledge demonstrates that cocaine causes distinct neuroadaptations for males and females^10^. The inclusion of both sexes within research investigating reward neurobiology is thus vital and necessary to the goal of treating addiction disorders. Future studies should include both males and females to understand how cocaine causes sex-specific neuroadaptations and for uncovering sexually dimorphic mechanisms that could lead to innovations for therapeutic intervention.

## Methods and materials

### Animals

Male and female mice on the C57BL/6 J background were used in this study. Mice were housed under a 12-h light–dark cycle at 23 °C with access to food and water ad libitum. Note that in compliance with ethical standards to minimize the use of mice, the mice used in this study were cre-negative offspring of *R26-CAG-LSL-Sun1-sfGFP;*A2a-cre and *LSL-Sun1-sfGFP*;Drd1-cre, generated for a separate study. All animal procedures were conducted in accordance with the National Institutes of Health Guidelines as well as the Association for Assessment and Accreditation of Laboratory Animal Care. Ethical and experimental considerations were approved by the Institutional Animal Care and Use Committee of The University of Pennsylvania. All experiments complied with ARRIVE guidelines.

### Investigator-administered cocaine

Male and female mice were handled daily for three days prior to cocaine administration. Following handling, mice were given a daily cocaine hydrochloride intraperitoneal injection (20 mg/kg dissolved in 0.9% saline) or 0.9% saline injection for 10 days^38^. Saline injected mice were used as the control group in all experiments. Male and female mice were injected with cocaine at the same time. Mice were sacrificed one day after the final injection. STR, HPC, and PFC tissue was immediately collected from each animal (Fig. 1A,B) and stored at − 80 °C until processing.

### S3EQ nuclear and cytosolic fraction separation

S3EQ^45^ was conducted as previously published with modified buffer volume. Tissue samples were homogenized in 350 μL cell lysis buffer (10 mM Tris–HCl (pH 8.0), 10 mM NaCl, 3 mM MgCl2 and 0.5% NP-40 in H_2_O) and spun for five minutes (1500 × g, 4 °C). The nuclei-containing pellet and RNA-containing cytosolic supernatant were separated and subjected to sequential ChIP or RNA extraction, respectively (Fig. 1C).

### Single and sequential chromatin immunoprecipitation (ChIP) and ChIP-PCR (qCHIP)

Prior to initiating the protocol, the following solutions were prepared as previously described^45,46^: BSA Blocking Buffer (0.5% Bovine serum albumin in 1 × PBS), Dilution Buffer (16.7 mM Tris–HCl (pH 8.0), 1.1% Triton-X 100, 0.01% SDS, 167 mM NaCl, 1.2 mM EDTA in H_2_O), Nuclear Lysis Buffer (50 mM Tris–HCl (pH 8.0), 5 mM EDTA, 1% SDS in H_2_O), Wash Buffer 1 (20 mM Tris–HCl pH 8.0, 150 mM NaCl, 2 mM EDTA, 1% Triton X-100, 0.1% SDS in H_2_O), Wash Buffer 2 (20 mM Tris–Cl pH 8.0, 500 mM NaCl, 2 mM EDTA, 1% Triton X-100, 0.1% SDS in H_2_O), Wash Buffer 3 (250 mM LiCl, 10 mM Tris–HCl pH 8.0, 1% sodium deoxycholate, 1 mM EDTA, 1% IGEPAL CA-630 in H_2_O), TE Buffer (10 mM Tris–HCl pH 8.0, 1 mM EDTA in H_2_O), Elution Buffer (0.1 M NaHCO_3_, 1% SDS in H_2_O).

M280 Sheep anti-Rabbit Dynabeads (Invitrogen, 11204D) were washed three times in BSA blocking buffer. The beads were bound to either H3K4me3 antibody (Rabbit Polyclonal, EMD Millipore, 07–473) or H3K27me3 antibody (Rabbit Polyclonal, EMD Millipore, 07–449) in 2X bead volume of dilution buffer and rotated for six hours (4 °C). Nuclei obtained from S3EQ were resuspended in PBS and fixed with 1% formaldehyde for eight minutes (350 rpm, 22 °C). 100 μL 1 M glycine was added and samples were rocked for five minutes to stop cross-linking (500 rpm, 22 °C). Samples were then centrifuged for five minutes (5500 × g, 4 °C) and the supernatant was discarded. The pellet was resuspended in 200 μL nuclear lysis buffer, transferred to TPX tubes, (Diagenode, C30010010) and incubated on ice for ten minutes. Samples were then sonicated in a Bioruptor^®^ (Diagenode) for two runs (high setting, 30 s on, 30 s off, 15 cycles). Dilution buffer was added to reach 1000 μL. 10% input (relative to each IP) was collected from each sample for normalization later used in qChIP analysis. The remaining diluted chromatin was combined with antibody-bound beads (1.2 μL bead-antibody slurry/1 μL chromatin), dilution buffer, and placed on a rotator (O/N, 4 °C). Samples were then washed with 1 mL of ice-cold Wash Buffer 1, Wash Buffer 2, Wash Buffer 3, and TE Buffer. Samples were rotated for five minutes during each wash (RT). Following washes, samples were rocked with 200 μL of elution buffer for 20 min (500 rpm, RT), centrifuged for three minutes (14,000 × g, RT), and placed on a magnet. The supernatant from each sample was divided in half and transferred to fresh tubes, each containing 100 μL of elution buffer. One tube was collected as the single ChIP sample.

The remaining tube was subjected to a second immunoprecipitation (sequential ChIP samples), in which each sample was subjected to antibody-bound beads that were bound to the reciprocal antibody (i.e., H3K4me3 ChIP samples were bound to H3K27me3 antibody-bound beads) and placed on a rotator (O/N, 4 °C). The sequential ChIP samples were processed according to the single ChIP protocol above. Single and sequential ChIP samples were then incubated for 4 h (65 °C) following the addition of 8 μL 5 M NaCl. Proteinase K (0.002 mg) was added, and incubation continued for two hours (65 °C). Samples were heat inactivated (15 min, 78 °C) and DNA was purified using the QiaAmp DNA Micro kit (Qiagen, 56304). qChIP was conducted as previously published^38^. Briefly, qChIP was run using Power SYBR™ Green PCR Master Mix (Life Technologies). The primer sequences that span − 100 to + 100bps of target promoter regions used to assess the presence of HPTMs^38^ are noted in Supplemental Table 1.

### RNA Extraction and qPCR

The cytosolic fraction following S3EQ was mixed with 600 μL RLT buffer (Qiagen) and RNA was extracted using the RNAeasy Micro Kit (Qiagen, 74004). RNA concentration was obtained using a Qubit 4 Fluorometer and RNA HS Assay Kit (Invitrogen). cDNA was synthesized using the Bio-Rad iScript™ cDNA Synthesis Kit (Bio-Rad, 1708890). qPCR was conducted using Power SYBR™ Green PCR Master Mix (Life Technologies). The primer sequences used to assess mRNA levels are noted in Supplemental Table 2.

### Pearson correlation analysis of qCHIP and qPCR

Pearson correlation analysis was used to assess the relationship between K4&K27 bivalent *Nr4a1* promoter, *Nr4a1* or *Cartpt* mRNA levels, and *Nr4a1* or *Cartpt* monovalent H3K4me3 or H3K27me3. Scripts were written and executed in R v4.1.1. First, all values corresponding to relevant endpoints were compiled for each sex (male, female) and brain region (STR, HPC, PFC) combination. For a given combination, the dataset was further separated based on treatment (saline, cocaine). Analyses were conducted with input values (fold change) from qChIP and qPCR data. An endpoint (qChIP or qPCR) was excluded if it lacked at least three observations in both the saline and cocaine datasets. An all-against-all Pearson’s correlation matrix was generated for the saline and cocaine datasets separately using the base R cor (method = ”pearson”) function. A correlation heatmap was generated from the Pearson correlation matrix using the corrplot() function from the corrplot package.

### Statistical analyses

Statistical tests used reflect the experimental design^75^. Specifically, while male and female mice were treated with cocaine in a single cohort, the tissue for each sex and each brain region was processed and analyzed separately due to the technical limitations associated with processing large numbers of sample. We therefore did not apply ANOVA to analyze sex or region differences. G*Power: Statistical Power Analyses Software^76^ (v3.1) was used to determine sample size. Final sample size was reduced during experimental execution (from n = 10 to n = 8 per group) due to previous empirical experimental endpoints and technical limitations. The Grubbs test was used to identify all outliers after an entire data set was complete and before any other statistical tests were applied (alpha = 0.05). Student’s t-tests were used for qChIP and qPCR analyses as these experiments directly compared one factor (drug exposure) from two groups. Sex was not considered a variable within any analyses due to experimental execution. F-tests of variance were conducted in every analysis and Welch’s correction was utilized when outliers were removed and when variances differed between groups. These data are shown as mean ± SEM. Graphpad Prism V9.3 was used for qChIP and qPCR analyses. qChIP data was analyzed by comparing CT values of each experimental group versus the control group following normalization of each sample’s input using the **∆∆**Ct method^77,78^. Bivalency at *Cartpt* was not assessed, as not all sequential-immunoprecipitated samples reached minimum signal intensity for qChIP (CT values were ≥ 38). qPCR data was analyzed by comparing CT values of each experimental group versus the control group following normalization of a housekeeping gene (*Gapdh*) using the **∆∆**Ct method^77,78^. All experiments were carried out one to two times, and data replication was observed in all instances of repeated experiments. Additionally, every qChIP and qPCR dataset had a technical replicate conducted by an independent investigator to ensure the validity of the data. All data shown in final analysis come from the first experiment of replicates, and the data specifically comes from the technical replicate conducted by the first author. Regarding visual data representation, each data point in all graphs represent one animal (Figs. 2, 3, 4, 5). For Pearson correlation analysis, the *p*-value matrix of all correlations corresponding to each comparison was extracted using the two-sided cor.mtest(method = ”pearson”) function from the corrplot package with a 95% confidence interval. This statistical test for Pearson’s product-moment coefficient was calculated following a t-distribution ($t=\frac{r\sqrt{\mathrm{n}-2}}{\sqrt{1-{r}^{2}}},$ where n = number of observations and r = calculated Pearson correlation coefficient) with degrees of freedom equal to length (number of complete observations)–2. Heatmap tiles were shaded red or blue only when satisfying a *p* value ≤ 0.05, where the null hypothesis represents r = 0 (no correlation), using p.mat = p.mat_for_comparison and sig.level = 0.05 within the corrplot() function. Heatmap tiles were shaded gray when analysis was not conducted, as analysis would violate the rule of independence^79^. All scripts are available at: (https://github.com/HellerLAbeats/Nr4a1_bivalency).

## Supplementary Information

Supplementary Information.

## Data Availability

Raw data or further methodological information from the current study are available upon reasonable request from the corresponding author.
