# Supplementary material for: Cocaine regulation of Nr4a1 chromatin bivalency and mRNA in male and female mice
Source: Sci Rep. 2022 Sep 21;12:15735. doi: 10.1038/s41598-022-19908-9 (PMC9492678; doi:10.1038/s41598-022-19908-9)
Supplement: Supplementary file 1 — Supplementary Information. [file 41598_2022_19908_MOESM1_ESM.docx]

Supplementary Tables

“Cocaine regulation of *Nr4a1* chromatin bivalency and mRNA in male and female mice.” Delaney K. Fischer, Keegan S. Krick, Chloe Han, Morgan T. Woolf, Elizabeth A. Heller

| **Promoter Region** | **Forward Sequence** | **Reverse Sequence** |
| --- | --- | --- |
| Nr4a1 | ATTTACAACACCCCCTCCTCC | TTCCATTGACGCAGGGAGCG |
| Cartpt | ACACAAGAGCCGTCAATTCCA | TCGAGTTCCCAACACCGC |

**Supplementary Table 1.** Primer sequences used in qChIP.

| **Gene Name** | **Forward Sequence** | **Reverse Sequence** |
| --- | --- | --- |
| Nr4a1_Exon4/5 | ATGCCTCCCCTACCAATCTTC | CACCAGTTCCTGGAACTTGGA |
| Nr4a1_Exon6/7 | AGCTTGGGTGTTGATGTTCC | AATGCGATTCTGCAGCTCTT |
| Cartpt | ACGAGAAGGAGCTGATCGAA | TCTCTGAGGGGAACGCAAAC |
| A2A | ACTCTCCCCTCCACACCC | CATAGTTTCTGTCTTCCAGCCC |
| Drd1 | TTCTTCCTGGTATGGCTTGG | GCTTAGCCCTCACGTTCTTG |
| Drd2 | TGGACTCAACAACACAGACCAGAATG | GATATAGACCAGCAGGTTGACGATGA |
| Gapdh | AGGTCGGTGTGAACGGATTTG | TGTAGACCATGTAGTTGAGGTCA |

**Supplementary Table 2.** Primer sequences used in RT-qPCR.

| **Sex** | **Region** | **Drug condition** | **Relationship** | **Corr.** | **Corresponding Figures** |
| --- | --- | --- | --- | --- | --- |
| M | STR | Saline | *Nr4a1* mRNA (Exon 6/7) – *Nr4a1* H3K27me3/H3K4me3 | - | 2A, 2E |
| M | STR | Saline | *Nr4a1* H3K4me3 – *Nr4a1* H3K27me3 | + | 2B, 2C |
| M | STR | Saline | *Nr4a1* H3K4me3 – *Cartpt* H3K4me3 | + | 2B, 3B |
| M | STR | Cocaine | *Nr4a1* H3K4me3 – *Cartpt* H3K4me3 | + | 2B, 3B |
| M | STR | Saline | *Nr4a1* (Exon 4/5) – *Nr4a1* (Exon 6/7) | + | 2A |
| M | STR | Cocaine | *Nr4a1* (Exon 4/5) – *Nr4a1* (Exon 6/7) | + | 2A |
| M | STR | Cocaine | *Cartpt* mRNA – *Cartpt* H3K27me3 | + | 3A, 3C |
| M | STR | Cocaine | *Cartpt* mRNA – *Nr4a1* H3K4me3/H3K27me3 | + | 3A, 2D |
| M | STR | Cocaine | *Cartpt* H3K27me3 – *Nr4a1* H3K4me3/H3K27me3 | + | 3C, 2D |
| F | STR | Saline | *Nr4a1* mRNA (Exon 4/5) – *Cartpt* H3K27me3 | + | 4A, 5C |
| F | STR | Saline | *Nr4a1* mRNA (Exon 6/7) – *Cartpt* H3K27me3 | + | 4A, 5C |
| F | STR | Saline | *Nr4a1* (Exon 4/5) – *Nr4a1* (Exon 6/7) | + | 4A |
| F | STR | Cocaine | *Nr4a1* (Exon 4/5) – *Nr4a1* (Exon 6/7) | + | 4A |
| F | STR | Cocaine | *Nr4a1* H3K4me3 – *Cartpt* H3K4me3 | + | 4B, 5B |
| F | STR | Cocaine | *Nr4a1* H3K27me3 – *Cartpt* H3K4me3 | + | 4C, 5B |

**Supplementary Table 3.** Significant relationships in the Striatum (STR) defined by Pearson’s Correlation Matrices from RT-qPCR and qChIP data.

| **Sex** | **Region** | **Drug condition** | **Relationship** | **Corr.** | **Corresponding Figures** |
| --- | --- | --- | --- | --- | --- |
| M | HPC | Saline | *Nr4a1* mRNA (Exon 4/5) – *Nr4a1* H3K27me3 | - | 2F, 2H |
| M | HPC | Saline | *Nr4a1* mRNA (Exon 4/5) – *Cartpt* H3K4me3 | - | 2F, 3E |
| M | HPC | Cocaine | *Nr4a1* (Exon 4/5) – *Nr4a1* (Exon 6/7) | + | 2F |
| M | HPC | Cocaine | *Nr4a1* mRNA (Exon 4/5) – *Cartpt* H3K4me3 | + | 2F, 3E |
| M | HPC | Cocaine | *Cartpt* mRNA – *Nr4a1* H3K27me3/H3K4me3 | + | 3D, 2J |
| F | HPC | Saline | *Nr4a1* H3K4me3 – *Cartpt* H3K4me3 | + | 4G, 5E |
| F | HPC | Saline | *Nr4a1* H3K27me3– *Cartpt* H3K27me3 | + | 4H, 5F |
| F | HPC | Cocaine | *Nr4a1* H3K27me3– *Cartpt* H3K27me3 | + | 4H, 5F |
| F | HPC | Cocaine | *Nr4a1* (Exon 4/5) – *Nr4a1* (Exon 6/7) | + | 4F |
| F | HPC | Cocaine | *Cartpt* mRNA –*Cartpt* H3K4me3 | - | 5D, 5E |
| F | HPC | Cocaine | *Nr4a1* (Exon 4/5) – *Cartpt* H3K4me3 | + | 4F, 5E |
| F | HPC | Cocaine | *Nr4a1* H3K27me3– *Cartpt* H3K4me3 | + | 4H, 5E |

**Supplementary Table 4.** Significant relationships in the Hippocampus (HPC) defined by Pearson’s Correlation Matrices from RT-qPCR and qChIP data.

| **Sex** | **Region** | **Drug condition** | **Relationship** | **Corr.** | **Corresponding Figures** |
| --- | --- | --- | --- | --- | --- |
| M | PFC | Saline | *Nr4a1* mRNA (Exon 4/5) – *Nr4a1* H3K4me3/H3K27me3 | + | 2K, 2N |
| M | PFC | Saline | *Nr4a1* H3K4me3 – *Cartpt* H3K4me3 | + | 2L, 3H |
| M | PFC | Cocaine | *Nr4a1* H3K4me3 – *Cartpt* H3K4me3 | + | 2L, 3H |
| M | PFC | Cocaine | *Nr4a1* H3K4me3/H3K27me3 – *Nr4a1* H3K27me3/H3K4me3 | + | 2N, 2O |
| M | PFC | Cocaine | *Nr4a1* (Exon 4/5) – *Nr4a1* (Exon 6/7) | + | 2K |
| M | PFC | Cocaine | *Nr4a1* H3K4me3/H3K27me3 – *Cartpt* H3K4me3 | + | 2N, 3H |
| M | PFC | Cocaine | *Nr4a1* H3K4me3 – *Cartpt* H3K4me3 | + | 2L, 2H |
| F | PFC | Saline | *Nr4a1* mRNA (Exon 6/7) - *Nr4a1* H3K4me3/H3K27me3 | + | 4K, 4N |
| F | PFC | Saline | *Nr4a1* (Exon 4/5) – *Nr4a1* (Exon 6/7) | + | 4K |
| F | PFC | Cocaine | *Nr4a1* (Exon 4/5) – *Nr4a1* (Exon 6/7) | + | 4K |
| F | PFC | Cocaine | *Nr4a1* (Exon 4/5) – *Nr4a1* H3K27me3/H3K4me3 | - | 4K, 4O |
| F | PFC | Cocaine | *Nr4a1* (Exon 6/7) – *Nr4a1* H3K27me3/H3K4me3 | - | 4K, 4O |
| F | PFC | Cocaine | *Nr4a1* (Exon 4/5) – *Cartpt* mRNA | - | 4K, 5G |
| F | PFC | Cocaine | *Cartpt* mRNA – *Nr4a1* H3K4me3/H3K27me3 | + | 5G, 4N |

**Supplementary Table 5.** Significant relationships in the Prefrontal Cortex (PFC) defined by Pearson’s Correlation Matrices from RT-qPCR and qChIP data.
